# Monoclonal antibody applications in travel medicine

**DOI:** 10.1186/s40794-023-00212-x

**Published:** 2024-01-15

**Authors:** Hanna K. de Jong, Martin P. Grobusch

**Affiliations:** 1 Centre of Tropical Medicine and Travel Medicine, Department of Infectious Diseases, Amsterdam University Medical Centers, Location AMC, Amsterdam Infection and Immunity, Amsterdam Public Health, University of Amsterdam, Meibergdreef 9, 1105 AZ Amsterdam, The Netherlands; 2 Institute of Tropical Medicine & Deutsches Zentrum Für Infektionsforschung, University of Tübingen, Tübingen, Germany; 3 Centre de Recherches Médicales, (CERMEL), Lambaréné, Gabon; 4 Masanga Medical Research Unit (MMRU), Masanga, Sierra Leone; 5 Institute of Infectious Disease and Molecular Medicine, University of Cape Town, Cape Town, South Africa

**Keywords:** Monoclonal antibodies, Tropical infectious disease, Prophylaxis, Malaria, Travelers

## Abstract

For decades, immunoglobulin preparations have been used to prevent or treat infectious diseases. Since only a few years, monoclonal antibody applications (mAbs) are taking flight and are increasingly dominating this field. In 2014, only two mAbs were registered; end of October 2023, more than ten mAbs are registered or have been granted emergency use authorization, and many more are in (pre)clinical phases. Especially the COVID-19 pandemic has generated this surge in licensed monoclonal antibodies, although multiple phase 1 studies were already underway in 2019 for other infectious diseases such as malaria and yellow fever. Monoclonal antibodies could function as prophylaxis (i.e., for the prevention of malaria), or could be used to treat (tropical) infections (i.e., rabies, dengue fever, yellow fever). This review focuses on the discussion of the prospects of, and obstacles for, using mAbs in the prevention and treatment of (tropical) infectious diseases seen in the returning traveler; and provides an update on the mAbs currently being developed for infectious diseases, which could potentially be of interest for travelers.

**Supplementary Information:**

The online version contains supplementary material available at 10.1186/s40794-023-00212-x.

## Introduction

There is a steadily increasing interest in monoclonal antibodies (mAbs) to prevent and treat infectious diseases both as endemic and imported conditions. This review focuses exclusively on the aspect of applications in travel medicine, and not on potential use in disease-endemic settings. A few years ago, only two mAbs were registered; in 2023, more than ten mAbs are registered or have been granted emergency use authorization [1]. Not least due to the coronavirus disease 2019 (COVID-19) pandemic, mAbs have been put into the spotlight; although multiple phase 1 studies were already underway in 2019 for other infectious diseases, such as malaria and yellow fever [2–4]. Monoclonal antibodies (i) could be applied prophylactically before traveling abroad (i.e., for the prevention of malaria), which is called passive immunization (in contrast to the active immunization by means of vaccination), or could be used (ii) as post-exposure prophylaxis for preventing active disease (e.g., rabies); or (iii) to treat manifest travel-acquired infections (dengue fever, yellow fever). The use of mAbs in travel medicine might have its benefits under specific circumstances when compared to standard vaccination and prophylaxis strategies. For example, using mAbs to prevent a *Plasmodium falciparum* infection, as recently demonstrated, would require only one single administration intravenously or intramuscularly before departure, inducing protective immunity lasting for at least 12 weeks without significant adverse effects, as compared to daily or weekly oral drug intake with gastro-intestinal or psychiatric adverse effects [2]. Other examples would be the prophylactic use of single-dose mAbs for hepatitis A, or yellow fever for immunocompromised travelers, who might be – although not necessarily though – unable to generate an adequate antibody response, or who should not be given live-attenuated vaccines (i.e., yellow fever vaccine) [5, 6]. Furthermore, successful effort has been put in the treatment of diseases with a high mortality and morbidity such as Ebola virus disease (EVD) and yellow fever using mAbs [3, 7]; and newer therapeutic options are being developed for rabies and dengue fever [8]/ (NCT04273217/NCT03883620). This review discusses the prospects of using mAbs for the prevention (pre- and post-exposure) and treatment of (‘tropical’) infectious diseases seen in travelers, and provides an update on the mAbs currently being developed against other infectious diseases, which could potentially be of interest for the field of travel medicine.

### Immunoglobulins administered for the prevention and treatment of infectious diseases

Immunoglobulins have been used for decennia as primary prophylaxis, as post-exposure prophylaxis (PEP), and as treatment of fulminant infections, severe toxin-mediated, auto-immune-mediated post-infectious complications, or chronic infections (Table 1). In travel medicine, it is rather common to administer hyper-immune globulins against hepatitis A Virus (anti-HAV) or hepatitis B Virus (anti-HBV) derived from human convalescent plasma for passive immunization when a traveler is unable to produce immunoglobulins due to an immunodeficiency, or when there is not sufficient time to become fully vaccinated before departure, or when too young to be vaccinated (children < 6 months of age). Post-exposure prophylaxis (PEP) HRIG (human rabies immunoglobulin), convalescent plasma therapy (CPT) against rabies, is well-known and widely used. Depending on the severity of the contact with the suspected rabid animal and the vaccination status of the patient prior to the bite, HRIG is advised by the World Health Organization (WHO) as PEP, and should be given within a short time frame to prevent infection [9]. In the past, there have been cases where convalescent plasma against rabies and EVD have been administered to prevent mortality, with mixed results [10, 11]. However, over the past decennia, synthetically derived mAbs have proven to be successfully targeting infectious diseases; and that they could potentially replace the human- and or animal-derived hyper-immune globulins, or hyper-immune sera.

**Table 1:** Overview of licensed immunoglobulins used in the prevention and treatment of infectious diseases

**Type**	**Trade name**	**Indication**	**Year licensed**	**Administration**
**Intravenous immunoglobulins**				
**Intravenous Ig (IVIG)**	**Asceniv®; Bivigam®; Carimune®; Flebogamma® DIF; Gammagard ®liquid, S/D; Gammaplex®; Gamunex-C®; Intratect®; Kiovig®; Nanogam®; Octagam®; Panzyga®; Privigen®**	**Primary immune deficiencies, other**	**Various (FDA and or EMA)**	**i.v.**
	**Cutaquig®; Cuvitru®; Gammanorm®; Hizentra®; HyQvia®; Evogam®; Vivaglobin® Xembify®**	**Primary immune deficiencies, other**	**Various (FDA and or EMA)**	**i.m./s.c.**
	**Strimvelis®;**	**ADA-SCID**	**2016 (EMA)**	**i.v.**
**Hyperimmune globulins**				
**Hepatitis A-Ig**	**HNIG; GamaSTAN® S/D; Beriglobin® P**	**PrEP and PEP hepatitis A virus**	**Various (FDA, EMA or other) **	**i.m.**
**Hepatitis B-Ig**	**Zutectra®; HepaGam B®; HyperHEP B®; Nabi-HB®; Hepatect® CP**	**PrEP and PEP hepatitis B virus**	**Various (FDA, EMA or other)**	**i.v. /i.m.**
**Varicella-zoster Ig**	**VARIZIG®; Zoster Ig-VF;**	**PEP Varicella Zoster Virus**	**Various (FDA, EMA or other)**	**i.m.**
**Tetanus Ig**	**HTIG; Tetagam® P; Tetanus Ig-VF IM; HyperTET® S/D**	**PEP tetanus**	**Various (FDA, EMA or other)**	**i.m.**
**Rabies Ig**	**HRIG; Berirab® P; Imogam® Rabies; Rabies-HT; Kedrab®; Hyperrab®**	**PEP rabies**	**Various (FDA, EMA or other)**	**s.c.**
**Cytomegalovirus Ig**	**Megalotect®; Cytogam®**	**PrEP cytomegalovirus**	**Various (FDA, EMA or other)**	**i.v.**
**Anthrax Ig**	**Anthrasil®**	**Treatment of inhalational anthrax**	**2015 (FDA)**	**i.v.**
**Botulin Ig**	**BabyBIG®**	**Treatment of infant botulism caused by toxin types A and B**	**2003 (FDA)**	**i.v.**
**Vaccinia Ig**	**none**	**Treatment of vaccinia, PEP variola**	**2010 (FDA)**	**i.v.**
**Hyperimmune sera**				
**Diphtheria antitoxin (equine)**	**DAT**	**Treatment of symptomatic diphtheria**	**unlicensed**	**i.v.**
**Botulism Antitoxin (equine)**	**Botulism Antitoxin Bivalent (Equine) Types A and B; BAT (Heptavalent (A, B, C, D, E, F, G)**	**Treatment of symptomatic botulism**	**2005 (FDA)/ 2013 (FDA)**	**i.v.**
**Monoclonal antibodies**				
**Palivizumab **	**Synagis®**	**PrEP RSV**	**1998 (FDA)/1999 (EMA)**	**i.m.**
**Raxibacumab**	**none**	**PEP, and treatment of anthrax**	**2012 (FDA)/2014 (EMA, os)**	**i.v.**
**Obiltoxaximab**	**Anthim®**	**PEP, and treatment of anthrax**	**2016 (FDA)/2020 (EMA)**	**i.v.**
**Bezlotoxumab**	**Zinplava®**	**PrEP C.** ***difficile***	**2016 (FDA)/2017 (EMA)**	**i.v.**
**Ibalizumab**	**Trogarzo®**	**Treatment of HIV-1**	**2018 (FDA)/2019 (EMA)**	**i.v.**
**Docaravimab/miromavimab**	**TwinRab™/RabiMabs™**	**PEP rabies**	**2019 (FDA, os)**	**s.c.**
**Atoltivimab/maftivimab/odesivimab**	**Inmazeb®**	**Treatment of Ebola**	**2018 (EMA, os); 2020 (FDA, os)**	**i.v.**
**Porgaviximab**	**Zmapp®**	**Treatment of Ebola**	**2015 (EMA os, 2021 withdrawn)**	**i.v.**
**Ansuvimab**	**Ebanga™**	**Treatment of Ebola**	**2020 (FDA)**	**i.v.**
**Casirivimab/Imdevimab**	**Ronapreve®**	**PrEP, PEP, treatment of COVID**	**2020 (FDA, 2022)/2021 (EMA)**	**s.c./i.v.**
**Sotrovimab**	**Xevudy**	**Treatment of COVID**	**2021 (FDA, 2022)/2021 (EMA)**	**i.v.**
**Bamlanivimab/etesevimab**	**none**	**PEP, treatment of COVID**	**2021 (FDA, 2022)**	**i.v.**
**Regdanvimab**	**Regkirona™**	**Treatment of COVID**	**2021 (EMA)**	**i.v.**
**Tixagevimab/cilgavimab**	**Evusheld™**	**PrEP COVID**	**2021 (FDA)/2022 (EMA)**	**i.m.**
**Tocilizumab**	**RoActemra®/Tyenne®**	**Treatment of COVID**	**2021(FDA)/2021(EMA); EMA(2023)**	**i.v./s.c.**
**Nirsevimab**	**Beyfortus®**	**PrEP RSV**	**2022 (EMA)**	**i.m.**

The table comprises of European Medicine Agency (EMA) and U.S. Drug and Food Administration (FDA) licensed immunoglobulins as per May 2023, which does not contain all marketed products

*ADA-SCID* adenosine deaminase severe combined immunodeficiency, *DAT* diphtheria antitoxin, *HBAT* Heptavalent botulism antitoxin, *RSV* respiratory syncytial virus, *C. difficile* Clostridioides difficile, *HIV-1* human immunodeficiency virus type 1, *COVID* corona virus disease 2019, *os* orphan drug status

### An introduction to monoclonal antibodies

#### Structure and function

Human antibodies are molecules generated by plasma cells or stimulated memory B cells following infection with a pathogen, or in response to vaccination. Immunoglobulins (Ig) are structured as Y-shaped heterodimers composed of two light chains of 25 kDa each, and two heavy chains of at least 50 kDa, depending on the Ig-isotype. Furthermore, the heavy and light chains, which are linked by multiple disulfide bridges and non-covalent interactions, vary in both the number of bridges and interactions [12]. Functionally, the two-fragment antigen-binding domains (Fabs) can bind and neutralize pathogens, and are linked to the crystallizable fragment (Fc) domain by a hinge region giving them more flexibility, thereby enabling them to strongly interact with any antigen. The Fc domain is able to mediate effector functions (antibody dependent cellular toxicity, complement-dependent cytotoxicity, and antibody-dependent phagocytosis) on various immune cells and complement protein C1q. It is able to bind to other proteins such as the Fcy receptors (FcyRs). The Ig-isotypes may vary depending on whether the gene segments (alpha, mu, gamma, epsilon or delta) recombine with the variable region, whereby each subclass specializes in the elimination of different types of pathogens. The IgG class is the main isotype in the blood and extracellular fluid and the IgG1 isotype is the mAb which has been used most as basis for the development of therapeutic mAbs used against infectious diseases [12, 13]. Strategies to identify human therapeutic mAbs for infectious diseases can be classified as either targeted – whereby the mAbs which bind to a specific antigen is directly isolated, or targeted agnostically – in which functional assays are performed on secreted immunoglobulins obtained from the supernatant of single cell cultures. More details on the function and strategies to develop mAbs are described in the review of Pantaleo et al. [12].

#### Monoclonal antibodies and their clinical use

**Fig. 1:**
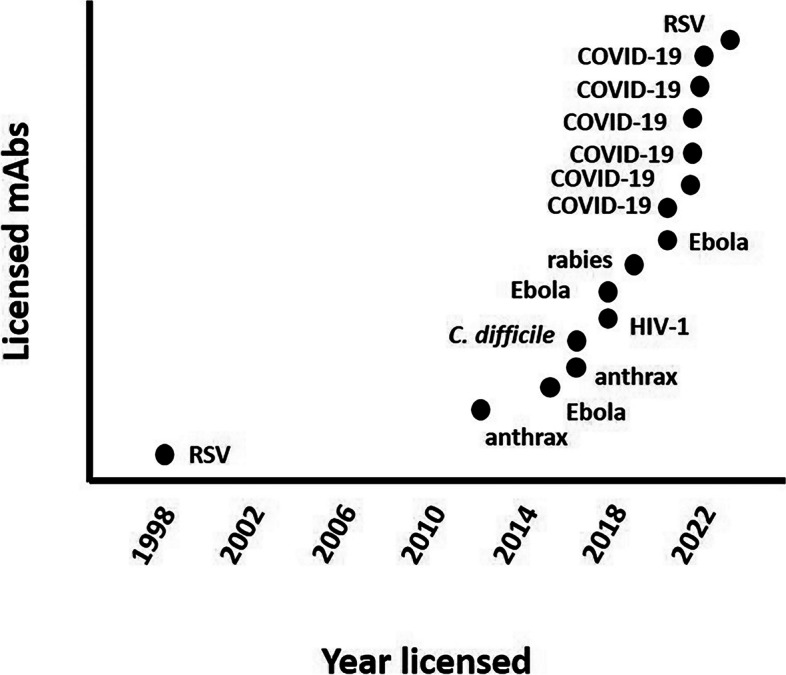
Expansion of number of licensed products for mAbs targeting infectious diseases over the last decades. Every single dot represents a licensed monoclonal antibody. On the x-axis, the monoclonal was licensed by either the FDA or EMA or both in the year indicated

Synthetically derived mAbs (from mouse or human cell lines) were first described in 1975 by Kohler and Milstein targeting sheep red blood cells [14, 15]. The first mAb registered in connection with an infectious disease was palivizumab (Synagis®, AstraZeneca) in 1998; which was developed as prophylactic agent against RSV infection in premature infants and infants with bronchopulmonary dysplasia [16]. Although multiple clinical trials for newer mAbs had already started prior to the COVID-19 pandemic, the number of registered mAbs for infectious diseases has grown exponentially (Fig. 1). The advantages of neutralizing mAbs compared to convalescent plasma therapy are numerous. Because they are synthetically derived, there is no risk of a blood-borne infection; the time to development of detectable high-affinity antibodies is shorter; molecules per unit are identical; availability does not depend on patient material and number of patients available, and there is no risk of low antibody titers which prevents inadequate pathogen neutralization. Furthermore, there is less chance of developing anaphylaxis (no relation with selective IgA-deficiency) or prion transmission. Lastly, due to molecular engineering, the half-life of mAbs could be prolonged compared to convalescent plasma therapy, and the potential risk of antibody-dependent enhancement (ADE) can be reduced by administrating large amounts of pathogen-specific antibodies and using plasma with high-affinity neutralizing antibodies [4, 17]. A potential disadvantage of mAbs could be the risk of loss of efficacy, as the mAbs are targeting a single specific epitope instead of convalescent plasma therapy, which could be derived from multiple donors, and which is therefore polyclonal. The latter, however, could be overcome by combining mAbs with different epitopes in order to create synergistic or additive effects [18]. Other disadvantages could be the risk of anaphylaxis or sensitization (which could be seen as an occupational hazard during drug handling). Of note, the costs of producing mAbs exceeds the production of vaccines, making them routinely available for high-income countries [19]. Moreover, fermentation tank production capacity is limited, thus rendering mass production difficult to envisage, if not impossible. For illustration, whereas mAbs are usually applied in microgram amounts per patients for non-infectious diseases indications, up to 10 g of mAbs might be needed, to treat an Ebola patient successfully [20, 21]. Below, we summarize relevant novel mAbs developed for infectious diseases and discuss their potential as primary prophylaxis, PEP and therapeutic options for travel medicine applications.

## Approach

For this scoping review, articles discussing mAbs with regard to infectious diseases treatment were searched and downloaded from the publicly available databases PubMed and Google Scholar. Registered immunoglobulin preparations for the prevention and treatment of infectious diseases (Table 1) were found on the publicly available website of the European Medicines Agency (EMA) and U.S. Food and Drug Administration (FDA), respectively; or found via public databases or websites of pharmaceutical companies producing the mAbs. Furthermore, articles of (pre-)clinical trials of unregistered mAbs targeting infectious diseases (Table 2) were searched and downloaded from PubMed using the key search terms: [diseases] AND [monoclonal antibody therapy]. As shown in Table 2, we focused our analysis on infectious diseases found amongst the top-10 diseases seen in returning travelers to Europe over the past two decennia as reported earlier [22] (see first column of Table 2 for the full list) excluding diseases with a predominantly self-limiting clinical course such as travelers’ diarrhea caused by viral infections [22]. Furthermore, in the section labeled as ‘other’, some diagnoses have been added, as these diseases could also be seen frequently in a travel clinic such as typhoid fever, leptospirosis, and more, and seem therefore to be relevant for this review. Articles having been published between 2013 and 2023 (October 21^st^) and deemed relevant to our focused topic, were included in this review. All relevant literature including original studies and clinical trials, were considered as long as their topic fell within the scope. Articles older than ten years, non-English abstracts, or preclinical studies with in vitro data only (without in vivo experiments), were excluded from this review. Regarding clinical trials involving mAbs, the registry clinicaltrial.gov was searched by the authors (October 21th 2023), and mAbs undergoing phase 1, 2, 3, and 4 clinical trials (Table 2) were included into this review. no studies

**Table 2:** Pipeline containing (pre) clinical studies on monoclonal antibodies targeting infectious diseases

Disease	Preclinical*	Phase 1	Phase 2	Phase 3	Phase 4
**Diarrheal disease**					
non-typhoid *Salmonella spp.*	**4**	**no studies**	**no studies**	**no studies**	**no studies**
* Shigella* spp	**1**	**no studies**	**no studies**	**no studies**	**no studies**
* Yersinia enterocolitica*	**no studies**	**no studies**	**no studies**	**no studies**	**no studies**
* Campylobacter jejuni*	**1**	**no studies**	**no studies**	**no studies**	**no studies**
* Vibrio cholerae*	**2**	**no studies**	**no studies**	**no studies**	**no studies**
Enterotoxigenic* E. coli*	**4**	**no studies**	**no studies**	**no studies**	**no studies**
* Entamoeba histolytica*	**no studies**	**no studies**	**no studies**	**no studies**	**no studies**
* Cryptosporidium spp.*	**1**	**no studies**	**no studies**	**no studies**	**no studies**
* Giardia lamblia*	**no studies**	**no studies**	**no studies**	**no studies**	**no studies**
* Cyclospora*	**no studies**	**no studies**	**no studies**	**no studies**	**no studies**
**Viral syndromes**					
Dengue	**15**	**AV-1**	**no studies**	**no studies**	**no studies**
		**DenguShield**			
Zika	**17**	**Tyzivumab**	**no studies**	**no studies**	**no studies**
		**DMAb-ZK190**			
Chikungunya	**23**	**mRNA-1944**	**no studies**	**no studies**	**no studies**
		**SAR440894**			
Japanese encephalitis	**2**	**no studies**	**ImmunoRel**	**no studies**	**no studies**
West Nile Virus	**4**	**MGAWN1**	**MGAWN1**	**no studies**	**no studies**
Tick-borne encephalitis	**3**	**no studies**	**no studies**	**no studies**	**no studies**
Rift Valley fever	**4**	**no studies**	**no studies**	**no studies**	**no studies**
Yellow fever	**5**	**TY014**	**no studies**	**no studies**	**no studies**
Ebola Virus Disease	**47**	**mAb114 (Ansuvimab)**	**Ansuvimab**	**Ansuvimab**	**Ansuvimab**
		**REGN3470-3471–3479 (REGN-EB3)**	**REGN-EB3**	**REGN-EB3**	**REGN-EB3**
		**Ebola (03-AT-2017) (GamEMab)**	**Gamezumab (01-AT-2020)**	**Gamezumab**	
			**Zmapp**	**Zmapp**	
Lassa	**3**	**no studies**	**no studies**	**no studies**	**no studies**
Marburg	**9**	**no studies**	**no studies**	**no studies**	**no studies**
Crimean Congo HF	**3**	**no studies**	**no studies**	**no studies**	**no studies**
Hanta virus	**6**	**no studies**	**no studies**	**no studies**	**no studies**
Hepatitis A	**no studies**	**no studies**	**no studies**	**no studies**	**no studies**
Hepatitis B	**10**	**Lenvervimab**	**Envafolimab**	**no studies**	**no studies**
		**HH-006**	**FG-3019**		
		**HH-003**	**Cetrelimab**		
		**HepB mAb19**	**HLX-10**		
		**IMC-I109V**	**HH-003**		
		**HepeX-B**			
Hepatitis C	**14**	**Bavituximab**	**anti-CD3**	**no studies**	**Tremelimumab**
		**MD11X06-02**	**MBL-HCV1**		
		**XTL6865**	**CT-011**		
		**CT-011**	**Anti-IL2R**		
Hepatitis E	**1**	**no studies**	**no studies**	**no studies**	**no studies**
Mpox	**4**	**no studies**	**no studies**	**no studies**	**no studies**
**Malaria**					
* Plasmodium* spp.	**14**	**CIS43LS**	**CIS43LS**	**no studies**	**no studies**
		**L9LS**	**L9LS**		
		**TB31F**			
		**MAM01**			
		**Meplazumab**			
**Rabies**					
* Rabies virus*	**8**	**CL184**	**CL184**	**SII RMAb**	**SII RMAb**
		**SII RMAb**	**SII RMAb**	**SYN023**	
		**SYN023**	**SYN023**	**GR1801**	
		**Rabies mAb CBB1**	**Docaravimab/ miromaviab**	**Docaravimab/** **miromavimab**	
			**Ormutivimab**	**Ormutivimab**	
**Trypanosomiasis**					
* Trypanosomia cruzi*	**5**	**no studies**	**no studies**	**no studies**	**no studies**
**Schistosomiasis**					
* Schistosoma spp.*	**1**	**no studies**	**no studies**	**no studies**	**no studies**
**Tuberculosis**					
* Mycobacterium tuberculosa*	**3**	**no studies**	**Pascolizumab**	**no studies**	**no studies**
**Other**					
Leptospirosis	**1**	**no studies**	**no studies**	**no studies**	**no studies**
Typhoid fever	**1**	**no studies**	**no studies**	**no studies**	**no studies**
Melioidosis	**1**	**no studies**	**no studies**	**no studies**	**no studies**
Rickettsioses	**no studies**	**no studies**	**no studies**	**no studies**	**no studies**
Strongyloidiasis	**no studies**	**no studies**	**no studies**	**no studies**	**no studies**
Leishmaniasis	**6**	**SCH708980**	**SCH708980**	**no studies**	**no studies**

*Preclinical studies: number indicates articles published before 21 of October 2023 (Pubmed) containing in vivo data on pipeline monoclonal antibodies. Clinical phases 1,2,3,4 also contain unpublished studies found on the registry clinicaltrial.gov (up until 21 of October 2023). Crimean *Congo HF* Crimean Congo hemorrhagic fever

## Literature review on the development of monoclonal antibodies with potential travel medicine applications

### Diarrheal disease

#### Acute diarrheal disease

Acute diarrheal disease is quite common among travelers both during, or shortly after their return, and was diagnosed in 9.3% of the evaluated ill travelers when presenting with symptoms to a EuroTravNet clinic between 1998 and 2018 [22]. Most disease courses are generally mild, self-limiting and most often do not necessitate use of any prescription drugs such as antibiotics; although in some cases, the condition could progress to dysentery and even toxic megacolon. Bacteria are regarded as the most predominant enteropathogens and account for most of the cases seen in travel clinics. Common pathogens cultured or find via PCR in stool of travelers are non-typhoid *Salmonella (S.) spp.*, *Shigella* spp*., Yersinia enterocolitica, Campylobacter jejuni,* enterotoxigenic *Escherichia (E.) coli*, and in rare cases *Vibrio (V.) cholerae*. Over the past years, there have been some publications on mAbs targeting these bacteria especially *S. typhimurium* and *V. cholera*, but none of these have entered the clinical trial phase thus far (Table 2). Viruses such as astrovirus, norovirus and rotavirus, are also known to cause acute travelers’ diarrhea but are generally self-limiting in adults. Acute diarrheal disease could also be caused by protozoal parasites such as *Entamoeba histolytica* and *Cryptosporidium* spp.*,* although only the latter has targeting mAbs in the preclinical phase (Supplementary file).

#### Chronic or persistent diarrheal disease

Persistent or chronic diarrhea is also in the top-10 diagnoses seen in travelers or migrants presenting with symptoms to a travel clinic [22]. Parasites are most often isolated from these patients, although some bacteria are known to cause persistent symptoms such as enteroaggregative or enteropathogenic *E. coli* or *Clostridioides (C.) difficile*. For the latter, bezlotuxumab, a fully human mAb which binds to *C. difficile* toxins A and B, is used as pre-exposure prophylaxis (PrEP) for patients with recurrent *C. difficile* infections but generally not used in the travel medicine setting (Table 1). The risk of a traveler of acquiring a protozoal infection rather than a bacterial infection increases with the duration of symptoms. Giardia is the most likely parasitic pathogen to cause persistent symptoms, which may last for months if left untreated. Other protozoal pathogens such as *Cryptosporidium* spp*., Cyclospora*, and *Entamoeba histolytica* are also found via PCR in stool of these patients. However, of almost all of the abovementioned pathogens none have targeted mAbs in the clinical stages thus far.

### Acute viral syndromes

Most of the currently licensed mAbs which are used as (preventive) treatment strategies are targeting viral infectious diseases (Table 1). Since the emergence of COVID-19 in 2019, there have been six licensed mAbs targeting SARS-CoV-2. Before COVID-19, there were only four licensed mAbs, targeting a variety of viral infections including RSV, HIV-1, rabies, and EVD [18]. Viral syndromes were also part of the top-three diagnoses seen in the returning travel presenting with illness [22]. When searching for mAbs targeting viral infections, a wealth of (pre)clinical studies was identified, mainly targeting viruses that yield the highest disease burden due to their virulence (i.e., EVD, rabies), due to high prevalence (i.e., hepatitis B and C) or high incidence (i.e., dengue, Zika, chikungunya) (Table 2). In Table 2, the number of published articles of preclinical studies which includes in vivo data is presented, and whose corresponding PMID identifiers can be found in the Supplementary file. Due to the wealth of studies including in vitro data only (especially on finding conserved epitope bindings site with potential high immunity) without evident clinical perspective, only in vivo (human and or animal) studies have been included in Table 2. For diseases such as tick-borne encephalitis, Rift Valley fever, Lassa fever, Marburg virus disease, Crimean-Congo hemorrhagic fever, hantavirus disease, hepatitis A, hepatitis E and Mpox, only preclinical studies could be found but none of the potential mAbs progressed into a clinical trial trajectory. All mAbs targeting a viral disease and undergoing phase 1,2,3 or 4 clinical trials, however, are reviewed below.

#### Dengue

Dengue is a (sub) tropical arboviral disease with an exponentially increasing incidence worldwide [23], with estimates running up to 50% of the global population at risk, and dengue featuring now amongst the top-frequently established diagnoses in travelers returning with a febrile condition from endemic areas [22]. Most people only experience mild symptoms when infected with the dengue virus, although in some cases, patients could develop a hemorrhagic disease or shock syndrome. A risk factor for the development of severe disease is having immunity against different serotypes, heterologous antibodies, also called antibody-dependent enhancement (ADE). As the incidence is rising, the risk for travelers to get infected with a different serotype is also increasing. Currently, no specific treatment exists for dengue, although dengue vaccine development lately made quantum leap progress towards several vaccines entering late stages of development and registration [24, 25]. Development of ADE, a feared complication of dengue vaccination seen in earlier vaccine trials, continues to be a matter of concern. The most recent registered dengue vaccine TAK-003 (Qdenga®), which has been marketed since spring 2023, did not show any important safety risks yet, and is registered for the indication of prevention of (secondary) dengue in travelers [26]. Although this is very promising, the current FDA/EMA licensed vaccines are live-attenuated and cannot be administered to pregnant or immunocompromised individuals. Due to the high incidence and potential progression to severe disease research on broadly protective antibodies, for instance targeting the flavivirus NS1 protein, are underway [27] (Table 2 and Supplementary file). When targeting the NS1 binding site, the risk of ADE is reduced as this is mainly seen when targeting the E protein, and the highly conserved NS1 epiptope can achieve flavivirus (dengue virus serotypes 1 to 4, yellow fever virus, Zika virus, West-Nile virus) cross-protection [28]. Two phase 1 studies with mAbs targeting dengue (AV-1 and Dengushield) have been completed but at the time of writing, results have not been reported yet in the peer-reviewed literature, or in the clinical trials registry (NCT04273217/NCT03883620).

#### Zika

From 2015 onwards, Zika virus disease (ZVD), moving eastwards through the peri-equatorial Pacific region, swept through the Americas; also, naturally, with implications for travelers [29, 30]. Although the risk of chronic morbidity was low and in relation to overall patient numbers, few deaths in adults were reported. The biggest threats arose from an increase in babies born with microcephaly during this epidemic (mainly in Brazil) due to mothers being infected especially during early pregnancy, a surge in Guillain-Barré Syndrome case numbers, an extremely rare but live-threatening immune-induced thrombocytopenia and overall, a risk of sexual transmission in the viremic phase [31, 32]. As there is no vaccine or treatment available, mAbs neutralizing Zika virus would be of great interest especially for pregnant women traveling to an endemic area. Two phase 1 studies have been registered to study the safety and tolerability of Tyzivumab, a single IV infusion mAb. One study was completed in 2018, but has not yet been published. The other, deemed phase 1 trial, has been withdrawn due to the decline in Zika virus cases (NCT03443830/ NCT03776695). Furthermore, a phase 1 trial has been set-up to evaluate the safety, tolerability and pharmacokinetic profile of DMAb-ZK190 in humans (NCT03831503). Synthetic DNA-encoded monoclonal antibodies (DMAbs) are an approach enabling in vivo delivery of DNA of highly potent mAbs to control infections via direct in vivo host-generated mAbs. The DMAb-ZK190, encodes for the mAb ZK190 neutralizing antibody, which targets the ZIKV E protein DIII domain, when in vivo-delivered, and achieved expression levels persisting > 10 weeks in mice and > 3 weeks in non-human primate, which is protective against Zika virus infectious challenge [33]. As discussed earlier, mAbs targeting the NS1 epitope seem to also protect against Zika virus replication in preclinical studies [28].

#### Chikungunya

Chikungunya virus (CHIKV), which is now prevalent in 110 countries worldwide, is an RNA virus in the *alphavirus* genus of the family *Togaviridae* and is transmitted by mosquitoes. Since 2004, outbreaks of chikungunya have become more frequent and widespread, and the incidence of chikungunya in returning travelers has since also increased [22]. CHIKV can cause a mild disease with fever, rash and arthralgia, but may also lead to a chronic polyarthritis in 50% of cases for which no cure exists [34]. Preclinical studies investigating mAbs in in vivo animal models seem promising (Supplementary file), for example in reducing the severity of CHIKV when administered to rhesus macaques [35]. In addition, another preclinical study showed that the use of CTLA4-Ig (Abatacept (Orencia®), registered for rheumatoid arthritis) provided partial clinical improvement (abolished swelling and markedly reduced levels of chemokines, pro-inflammatory cytokines, and infiltrating leukocytes) in a mouse model [36]. A phase 1 trial published in 2021 reports on the first mRNA-encoded mAb (mRNA-1944), showing in vivo expression and detectable ex vivo neutralizing activity against CHIKV in a clinical trial and may offer a potential treatment option for CHIKV infection [37]. The mRNA-1944 is a lipid nanoparticle-encapsulated messenger RNA encoding the heavy and light chains of a CHIKV-specific monoclonal neutralizing antibody, and, when intravenously administered, resulted in rapidly generated levels of neutralizing antibodies at all doses tested by 12 h that peaked within 48 h with a measured mean half-life of approximately 69 days. The high antibody levels achieved 36–48 h after infusion exceeded the target level of the protective CHIKV neutralizing antibody level of 1 µg mL^−1^, which has been shown previously to be associated with protection from both symptomatic chikungunya infection and subclinical seroconversion. No major safety issues have been reported, and this mRNA technology for protein production may reduce the need to deliver high doses of antibodies which are typically required for therapeutic antibodies. Further studies are needed to determine the duration of protection and efficacy of mRNA-1944. Another phase 1 trial studying the mAb SAR440894 in a single dose escalation study is currently underway (NCT04441905).

#### Japanese encephalitis

Japanese encephalitis virus (JEV) causes a vaccine-preventable febrile disease with an encephalitic picture in Asia and the western Pacific [38]. Especially during flooding, the incidence will increase and more people in endemic areas should be (re-)vaccinated. Several highly effective vaccines are brought to market over the past decades, classified in four classes; inactivated mouse brain-derived vaccines, inactivated Vero cell-derived, live attenuated, and live recombinant (chimeric) vaccines [39]. As the risk for infection for travelers is low, the vaccine is only given to travelers under specific circumstances (i.e., will stay for longer periods or when spending time in rural areas). For those patients developing neurological symptoms, no specific treatment is available, and the use of antibodies would be desirable. No clinical studies for the use of mAbs have been registered. There has only been one randomized double-blind placebo-controlled phase 2 clinical trial with IVIG containing anti-JEV neutralizing antibodies (ImmunoRel®), 400 mg/kg/day for 5 days) given to a limited number of children with suspected JE in Nepal [40]. Although the proportion of patients fully recovering (without any sequelae) was similar between the groups at discharge and slightly higher among patients in the IVIG group at follow, this difference was not significant on intention-to-treat analysis. As the number of patients included was low, the efficacy of ImmunoRel® can only be studied in a full phase 3 randomized placebo-controlled trial.

#### West Nile virus

West Nile virus (WNV) is a mosquito-borne *flavivirus* that has a bird–mosquito–bird transmission cycle where humans are a dead-end host. As WNV has spread rapidly over many continents including Europe and North-America, it is now one of the most widely distributed arboviruses worldwide [41]. Similar to JEV, in most cases, the infection with WNV is subclinical. Only in a small percentage will it lead to an encephalitis or meningitis with a potentially devastating outcome. Furthermore, long-term sequelae have been reported such as muscle weakness, memory loss, and difficulties with activities of daily living after infection with WNV, which could be a risk for travelers [41]. Currently, no vaccine is registered but as the incidence is increasing, therapeutic options available when severe (neurologic) symptoms do occur, would be most welcome. Not many preclinical studies have been published (Table 2 and Supplementary file). In humans, the safety and pharmacokinetics of a single dose of the iv-administered MGAWN1, a novel mAbs targeting the E protein of WNV, has been studied in a phase 1 trial [42]. A single iv infusion of saline or of MGAWN1 at escalating doses (0.3, 1, 3, 10, or 30 mg/kg of body weight) was administered to 40 healthy volunteers (30 receiving MGAWN1; 10 receiving placebo) and was well tolerated and no major safety concerns were reported. MGAWN1 had a half-life of 26.7 days and a maximum concentration in serum (C(max)) of 953 µg/mL, which exceeds the target level in serum estimated from hamster studies 28-fold, which is expected to yield neutralizing activity and penetration across the blood–brain barrier. A phase 2 study with MGAWN1 was started but has been early terminated due to the inability to enroll subjects (only 13 out of the 120 subjects estimated) (NCT00927953).

#### Yellow fever

Yellow fever is a primarily mosquito-transmitted disease affecting humans and non-human primates in tropical areas of Africa and South America. Due to the wildlife reservoir, eradication is almost impossible, but large-scale mass vaccination activities in Africa during the 1940s to 1960s reduced yellow fever incidence for several decades [43]. The yellow fever virus is known to cause an acute viral hemorrhagic disease with a mortality up to 20 to 50% especially when liver failure occurs. Imported cases in travelers are few, but devastating [22, 44]. The live-attenuated vaccine gives a high protection rate but is contra-indicated in infants, in pregnant women, people aged > 60 years, and the more severe immunocompromised hosts due to the risk of vaccine-induced viscerotropic and neurotropic serious adverse events [5]. Since there is no antiviral therapy nor cure once this disease manifests, studies looking at mAb therapy are ongoing (Supplement file). The first phase 1 trial studying the safety, side-effect profile, and pharmacokinetics of TY014, a fully human IgG11 anti-yellow fever virus mAb, was published in 2020 [3]. The half-life of TY014 ranged from 6.5 to 17.5 days among individual participants across the five dose cohorts (0.5–40 mg/kg), and no major safety concerns were reported. Both groups (placebo vs TY014 infused) received the YF17D live attenuated vaccine as a challenge virus. The subjects who received the mAb TY014 (2.0 mg/kg iv) were able to curb viremia and reduce the incidence of vaccine-induced symptoms. It also prevented the induction of innate immunity- and pro-inflammatory response genes, whose expressions are associated with a more severe outcome in yellow fever patients. Although no real infection challenge could be performed, these finding do suggest that the mAbs could interrupt yellow fever pathogenesis, and further studies are necessary to examine the prophylactic and post-exposure treatment potential of TY014.

#### Ebola virus disease

Ebola virus disease (EVD) is caused by various Ebola viruses (EBOV) within the genus *Ebolavirus*; with the closely-related Marburg virus (genus *Marburgvirus*), causing very similar disease in a comparable outbreak pattern [45]. EVD is known for its high mortality (case fatality rate of 50%) and may present itself with a hemorrhagic fever which could affect both humans and other primates. The virus can be contracted via blood, secretions, organs and other bodily fluids of infected people, and is transmitted by wild animals such as fruits bats, porcupines and non-human primates. The risk of infection for travelers is low as most infections occur in remote areas in sub-Saharan Africa; although during the 2014–2016 Western African EVD outbreak, there was a serious threat for people traveling to endemic areas (especially for health care workers) to get exposed to the virus [46]. There are currently two licensed vaccines by the EMA and FDA, the rVSV-ZEBOV (Ervebo®) and Ad26.ZEBOV/MVA-BN-Filo (Zabdeno®/Mavbea®), which both only targets the (Z)EBOV (or *Zaire ebolavirus*), while the most recent outbreak in Uganda was caused by the Sudan strain (Sudan virus or SUDV) [47]. Monoclonal antibody treatment of EVD, of which three have been licensed (of which one is already withdrawn) by the EMA and or FDA (Table 1), is methodologically well-established and technically amongst the most advanced in the field. However, mass application in a large-scale outbreak will remain difficult due to production logistics and cost and the risk that current mAbs might not be best suited for the then-outbreak-causative ZEBOV strain, let alone if an outbreak is caused by a non-ZEBOV EBV. The origins of ‘antibody therapy’ of Ebola in the broadest sense lie in the administration of convalescent plasma and full blood to Ebola patients. Very few anecdotal clinical data and some supporting animal data from the era prior to the West African outbreak 2013–2016 suggested that antibodies contained in convalescent full blood and plasma – all risks of transmitting infectious diseases taken into account – have the potential to prevent death and facilitate recovery of Ebola patients [48–50]. Further data on CPT are limited to very few cases, reviewed by Sullivan and Roback [51].

Even before the large West African EVD outbreak, more than twenty mAbs for the treatment of EVD had been identified and characterized, of which several were found promising to progress to testing in non-human primate models, as single antibodies or in combination [21]; in the meantime amounting to several hundreds have described the particular structure of mAbs targeting Ebola virus glycoprotein (GP) structures in relation to the specificities of the GP target in detail [52] (Supplementary file). In principle, mAbs bind to the GP which governs virus attachment and host membrane fusion [20]. Fausther-Bovendo and Kobinger as well as Pantaleo and colleagues recently reviewed the pre-clinical and clinical development of Ebola antibodies in much detail [12, 53]. In essence, the first key clinical trial, including patients recruited in all three afflicted West African countries, was a randomized controlled trial of the ZMapp mAbs cocktail plus the (symptomatic treatment) standard-of-care versus stand-of-care alone during the West African outbreak. ZMapp contains three chimeric antibodies (13C6, 4G7 and 2G4) as combined from earlier experimental combinations MB-003 and ZMab [20]. In the PREVAILII trial deaths were 8/36 (22%) of cases in the intervention group versus 13/35 (37%) in the standard-of-care-alone comparator group, with a post-hoc observed probability of 91% of superiority of the ZMapp-applying intervention arm, and an absolute difference in mortality of -15% in frequentist analyses (CI -36 to 7) and although ZMapp appeared to be beneficial, the pre-specified statistical efficacy threshold of 97.5% was not met [54]. The PREVAILII results informed the study design of the PALM trial. In the PALM trial conducted in the East Kivu outbreak which began in 2018, 681 patients were randomly assigned in a 1:1:1:1 ratio to four investigational regimes – ZMapp as control, remdesivir, Mab114 as single mAb and the REGN-EB3 triple mAbs cocktail consisting of three human mAbs REGN3470, -3479 and -3471. At Day 28, the percentage of patients who died was lower in the MAb114 group and in the REGN-EB3 group than in the ZMapp group which led to the withdrawal of ZMAPP as standard treatment [55]. The PALM trial results are up to date and are considered decisive with regard to the now current standard of care regarding (Z)EBOV outbreaks [56]; however, in the most recent SUDV in Uganda, the REGN-EB3 cocktail (Inmazeb®) as well as mAb114 (Ebanga®) are naturally ineffective. Currently clinical studies looking at other mAbs which could be used for the emergency prevention of Ebola Virus Disease are registered but have not been published (NCT03428347/NCT04717830). Of note, administered mAbs (Mab114 or REGN-EB3) to high and intermediate-risk contacts of EVD patients appear to be promising candidates to protect these contacts [57].

Regarding the closely related Marburg virus disease; with an increasing number but very small outbreaks usually coming to an early end up to now, none of the candidate mAbs (Table 2) could be put to the test in the field up to now.

#### Hepatitis B

Hepatitis B virus (HBV) is currently the main cause of chronic hepatitis worldwide, and is most commonly transmitted vertically (from mother to child during birth and delivery), through contact with blood or other body fluids during sex with an infected partner, unsafe injections or exposures to sharp instruments. Although the vaccine has a 100% protection rate, most people are not aware they carry the HBV and could infect unvaccinated people. The disease be suppressed with antiviral therapy, but not cured [58]. When left untreated, chronic HBV infection leads to end-stage liver cirrhosis and/or hepatocellular carcinoma (HCC). For pre- and post-exposure applications, several immunoglobulin preparations targeting HBsAg (anti-HBs/HBIG) may be used (Table 1), and research suggests they could also be used for treatment of HBV [59]. Many pre-clinical studies studying mAbs targeting different epitopes of HBV in several mouse models have been published (Table 2 and Supplementary file) [60]. Multiple clinical phase 1 studies have been registered with clinicaltrial.gov for mAbs targeting HBV (HH-006 (NCT05275465); HH-003 (NCT05542979), HepB mAb19 (NCT05856890); IMC-I109V (NCT05867056); HepeX-B (NCT00228592)), although only one study has been published in literature [61]. Lenvervimab is a recombinant human immunoglobulin used for the treatment of chronic HBV. HBV patients with a persistently positive serum HBsAg for at least six months were recruited for this open-label, dose-escalation phase 1 trial in which patients were given a single or weekly intravenous infection of lenvervimab (doses ranging from 80,000 to 240,000 IU) for four weeks. The primary endpoint was a decrease in HBsAg to less than the limit of quantitation without any rebound within one month but was only reached in two out of nine patients (22.2%) in the highest-dose group. No safety issues or dose-related toxicity was reported. The authors suggest this mAb might, in combination with a nucleoside analogue, lead to sustained clearance of HBsAg in patients with chronic HBV infection and is less allogenic and costly than plasma-derived HBIG. As mentioned in the article, a phase 2 study is underway which hopes to lead to a better understanding of how lenvervimab works in combination with antivirals. Other phase 2 trials are reported on clinicaltrial.gov but have not been published as of yet (envafolimab (NCT0446589), FG-3019 (NCT01217632), cetrelimab (NCT05242445), HLX-10 (NCT04133259), HH003 (NCT05861674/NCT05839639/NCT05734807/NCT05674448).

#### Hepatitis C

Hepatitis C virus (HCV) is a blood-borne virus with a high global burden, and most infections occur through exposure to infected blood via unsafe injection practices, unscreened blood transfusions, injection drug use, and sexual practices. In travel clinics, chronic disease is most often diagnosed in migrants during routine screening activities rather than acute illness episodes [62]. Although HCV could lead, if untreated, to liver fibrosis and end stage liver cirrhosis; in contrast to hepatitis B, there is a cure. A sustained virological response (SVR) is seen in 98% of patients with chronic HCV when treated with an oral direct-acting antiviral agent (DAA) combination regimen for 8—12 weeks [63]. Unfortunately, prevention of disease is not possible as there is no vaccine, and the antivirals have not been tested for use as PrEP. As with HBV, there seems to be a lot of interest in studies with mAbs, for HCV specifically targeting the HCV envelope, for curation and prevention of disease (Table 2). The challenge is to develop mAbs that are either at least as effective as the DAAs but with fewer adverse effects, or that, when combined with antiviral drugs, can circumvent long-term use of these drugs thereby reducing their side effects and augmenting their antiviral effect. Multiple phase 1 and 2 trials for mAbs targeting HCV are underway, of which some are published and some are registered on clinicaltrial.gov but have not been published by the time of writing (bavituximab (NCT00128271/ NCT00343525), XTL6865 (NCT00300807), CT-011 (NCT00962936); anti-IL2R B ab)). The mAb MBL-HCV1, targeting the HCV E2 glycoprotein, significantly delayed median time to viral rebound in patients with chronic HCV genotype 1a undergoing liver transplant compared to placebo treatment (18.7 days vs. 2.4 days, p < 0.001) in a double-blind, placebo-controlled trial [64]. Although monotherapy with MBL-HCV1 did not prevent allograft infection as antibody-treated subjects had resistance-associated variants at the time of viral rebound, further studies in combination with DAA’s are underway. The antiviral potential of another mAb, BMS-936558 (MDX-1106), a fully human anti-PD-1 monoclonal immunoglobulin-G4 that blocks ligand binding, was tested in a placebo-controlled single ascending dose study in patients with chronic HCV [65]. Persistent viremia, as seen in chronic hepatitis C patients, has been associated with the upregulation of PD-1 expression on virus-specific CD8 + T cells. In this proof-of-concept study, a single dose of BMS-936558, was generally well tolerated and led to HCV RNA reductions ≥ 0.5 log10 IU/mL in five of 45 (11.1%) patients and suppression of HCV replication persisted more than eight weeks in most patients. In a phase 2a clinical trial, the benefits of orally administered anti-CD3 mAb has been studied [66]. Orally administered anti-CD3 antibody exerts its effect mainly at the level of the gut-associated lymphoid tissue and mesenteric lymph nodes and exerts a systemic immune modulatory effect via promotion of specific T-cells. In this placebo-controlled trial, a 30-day course of oral anti-CD3 mAb immunotherapy was safe and well tolerated, and was associated with improvement in hepatic and immunologic parameters seen in patients with chronic HCV together with a reduction of HCV viral load. Particularly chronic HCV patients who are non-responders to antiviral therapy could potentially benefit from immune enhancement in the gut. Another mAb that is studied for its antiviral effect via immunomodulation is tremelimumab, a fully human IgG2 mAb that blocks the binding of cytotoxic T-lymphocyte-associated antigen 4 (CTLA-4), which has been registered for adult patients with metastatic non-small lung cancer by the FDA in 2022. In this phase 4 study, tremelimumab was administered at a dose of 15 mg/kg on day one of every 90-day cycle to patients with inoperable HCC and chronic HCV [67]. The therapy was well tolerated and showed both a reduction in the tumor load as well as in HCV viral load reduction. The authors suggest that the combination of this mAb together with DAAs is worth being explored in patients with interferon-resistant HCV infection.

### Malaria

Malaria is a preventable and curable but potentially life-threatening vector-borne disease caused by five *Plasmodium (P.)* spp. causing disease in humans. In particular, if left untreated, or treated late, *P. falciparum* leads to life-threatening disease particularly in the non-immune. Travelers going to high- and middle-endemic areas are advised to take malaria chemoprophylaxis [68]. Currently only one vaccine has been brought to the international market, RTS,S/AS01, but more are being in development with great expectation [69, 70]. In April 2023, the Ghanese Food and Drug Authority approved the R21/Matrix-M vaccine which has proven to have a higher efficacy than the RTS,S/AS01 vaccine. Currently, the WHO recommends the RTS,S/AS01 malaria vaccine only for children living in regions with moderate to high *P. falciparum* malaria transmission. The vaccine is not usable for travelers traveling to an endemic area due to its low efficacy. Single-dose mAbs used as malaria prophylaxis or as treatment are currently being extensively studied and most of the research is done on *P. falciparum* (Table 2 and Supplementary file). (Recently published articles on phase 1 and 2 trials of the mAbs targeting *P. falciparum* are summarized below.

The most abundant antigen on the sporozoite surface is the *P. falciparum* circumsporozoite protein (PfCSP), which is required for attachment to host hepatocytes. In 2021, a first-in-human, open-label, phase 1 dose-escalation clinical trial has been published by Gaudinsky et al*.*, showing promising results regarding the mAb developed to act directly against the P*f*CSP*.* The human mAb (CIS43) has been isolated from a human subject immunized with one of the Sanaria Inc. whole sporozoite vaccines [2]. The CIS43 binding specificity for the NPDP epitope, an important antigen target, seemed very effective in preclinical trials [71]. Furthermore, the mAb has been enhanced to increase the half-life from three weeks to longer-lasting immunity, to up to 36 weeks. Although the study has suffered from the COVID pandemic and therefore, following a protocol change, the end results show that among adults who had never had malaria infection or vaccination, a single-dose administration of the long-acting mAb CIS43LS with higher doses (20 mg/kg or 40 mg/kg i.v.) prevented malaria after controlled infection, and was well tolerated. Limitations were the small sample size and the absence of breakthrough infections; therefore, the threshold of CIS43LS could not be defined. Most recently, the third part of the phase 1 trial has been published, reporting the ability of CIS43LS to confer protection at lower doses intravenously administered (1 mg/kg, 5 mg/kg or 10 mg/kg) or by the subcutaneous route (5 mg/kg and 10 mg/kg). In this study, it is concluded that a single dose of CIS43LS at 5–10 mg/kg, administered subcutaneously or intravenously, provides high-level protection against controlled human malaria infection approximately 8 weeks (48–56 days) after antibody administration [72]. Studying the CIS43LS mAbs in a malaria-endemic area would shed further light on the usage of these monoclonal antibodies in travelers as substitute for malaria chemoprophylaxis, which has been published most recently [73]. In this randomized, dose escalating study, healthy adults in a malaria-endemic area were given a single intravenous dose of CIS43LS (10 or 40 mg/kg) or placebo over a six months malaria season in Mali. Every two weeks, thick smear examination was performed to study the primary efficacy endpoint. At six months, the efficacy of 40 mg of CIS43LS per kg as compared with placebo was 88.2%, and the efficacy of 10 mg of CIS43LS per kg bodyweight as compared to placebo was 75.0%. Although participants had a higher risk of moderate headache, CIS43LS was proven to be protective against *P. falciparum* infection during a 6-month malaria season in Mali and could be considered as an interesting alternative for travelers. Another potential mAb, L9, targeting a different conserved site in the junctional region of PfCSP appears to be two to three times more potent than CIS43. A phase 1 trial was recently published to assess the safety and pharmacokinetics of L9LS in healthy adults [74]. Both subcutaneous and intravenous administration were being tested with different doses (1 mg, 5 mg, or 20 mg/kg of body weight) followed by a controlled human malaria infection (*P. falciparum* 3D7 strain). Compared to the CIS43 mAb, the half-life extension was similar, with an estimated 56 days. Both the five- and 20 mg doses, administered intravenously, yielded 100% protection in the human malaria challenge model. To further study its potential, three phase 2 trials (NCT05304611/NCT05400655/ NCT05816330) are currently underway, studying L9LS in Mali involving children and adults, and in Kenya including infants [74]. Lastly, a phase 1 trial studying the mAb TB31F, that binds to the gametocyte surface protein Pfs48/45 and inhibits fertilization, thereby preventing further parasite development in the mosquito midgut and onward transmission, was recently published [75]. Malaria-naïve participants were administered a single intravenous dose (ranging 0.1 – 10 mg /kg) or subcutaneous dose of 100 mg TB31F, and were monitored for 84 days primarily for adverse events. Further analyses included TB31F serum concentrations and transmission-reducing activity (TRA) of participant sera. Administration of TB31F was well tolerated, did not lead to serious adverse events, and appeared to be a highly potent mAb capable of completely blocking transmission of *P. falciparum* parasites from humans to mosquitoes for a duration of 160 days. The latter means it could potentially block the transmission cycle for a complete malaria season. Currently, no mAbs targeting other malaria *species* pathogenic to man *(P. ovale* subspecies*, knowlesi, vivax or malariae)* have been tested in human clinical trials yet.

### Rabies

Rabies, caused by a neurotropic *Lyssavirus*, has a case-fatality rate of almost a 100%. When vaccinated, the immunological memory is reactivated within seven days after a single intramuscular booster immunization, even when administered 10–24 years after PrEP [76]. Once an unvaccinated human is being bitten by a – proven or suspected—rabid animal, PEP containing HRIG must be administered preferably within 48 h [77, 78]. However, HRIG is expensive and complex to produce, and a synthetically derived alternative would be ideal. Most cross-reactive mAbs developed for neutralizing the rabies virus are targeting the outer viral glycoprotein. The first to be used mAbs in humans were a cocktail of two, CR57 and CR 4098 (together called CL184), which were shown to be broadly neutralizing across many rabies virus isolates during pre-clinical research and were also tested in phase 1 and 2 trials. Although the safety and presence of rabies virus neutralizing antibodies in these studies seemed hopeful, the pharmaceutical company, for unknown reasons, decided to withdraw the mAb from further development [77]. The first mAb registered in humans was in 2016, was well tolerated, and was also directed against the rabies virus glycoprotein antigenic site III (SII RMab or Rabishield) [79]. SII RMab is currently licensed in India and was tested in a phase 2/3 trial, where it demonstrated to be non-inferior to standard HRIG in rabies-exposed individuals in India [80]. A further phase 4, multicenter, randomized, controlled study of the safety and immunogenicity in patients with potential rabies virus exposure is underway. The only concern which was raised for this mAb was that it did not neutralize all rabies variants and therefore the WHO has marked a slight risk for use in the Americas region [77]. The second mAb, docaravimab/miromavimab (Twinrab™ or Rabimabs™), which could be used as PEP, received orphan status by the FDA and was approved in 2019 (Table 1). Recently, a phase 2/3 trial was published and demonstrated non-inferiority after administration of 40 IU/kg Twinrab™ in safety and efficacy to standard 20 IU/kg HRIG in rabies virus exposed patients in India [81]. Three other mAbs are currently at the phase 2/3 stage namely SYN023, Ormutivimab, and GR1801. SYN023 consists of two humanized mAbs, CTB011 and CTB012, and was given to subjects in a phase 2 study in a single dose of 0.3 mg/kg in combination with five vaccine doses [82]. In this study, SYN023 provided adequate antibody coverage and treatment related adverse events were comparable to RIG. A phase 3 study has recently been completed (NCT04644484) but results have not been published by the time of writing. Ormutivimab, a mAb of the IgG1 subtype, is the third recombinant human anti-rabies mAb marketed and has been approved for PEP of rabies virus in China with a dose of 20 IU/kg. In a phase 2b trial conducted in China, healthy volunteers received 20 IU/kg, 40 IU/kg or 20 IU/kg HRIG in combination with vaccination [83]. The combination of ormutivimab and rabies vaccine induced higher neutralizing antibodies levels in the early stage and less interventions to the vaccine. The lower dosage seemed as effective with the least adverse events, therefore in the phase 3 confirmatory clinical study, the efficacy and safety of 20 IU/kg ormutivimab injection combined with rabies vaccine in class III exposed persons attacked by suspected rabies animals will be further explored. GR1801, a mAb indicated for PEP of WHO Category 3 rabies exposure patients has entered a phase 3 clinical trial and is currently recruiting (NCT05846568). Patients of the marketed mAbs for rabies are given as PEP, but none has been studied as potential cure once symptoms manifest. However, preclinical data published on mAbs as cure for rabies in mice do have potential [8].

### Trypanosomiasis and schistosomiasis

Chagas disease, caused by *Trypanosoma (T.) cruzi,* and schistosomiasis, caused by different *Schistosoma (S.)* spp., are both parasitic infections diagnosed in migrants and travelers presenting to the travel clinics for screening activities [22]. Chagas disease is especially difficult to treat once in the chronic stage, and could cause severe cardiomyopathy and death. Schistosomiasis can also persist for years and can lead to increased risk of liver fibrosis or bladder cancer. For both diseases, only preclinical studies have been published on mAbs. For Chagas disease, especially mAbs targeting TNF such as infliximab in animals infected with *T. cruzi* seems to positively impact on the severity of cardiac disease [84]. Bevacizumab, a monoclonal antibody that functions as an angiogenesis inhibitor, showed a regression in the vascular activity and microvascular density in mice infected with *S. mansoni *[85]. Currently none of the mAbs have entered the clinical phase.

### Tuberculosis

Tuberculosis, caused by *Mycobacterium tuberculosis*, is the most common bacterial infection seen in migrants [22]. For tuberculosis, especially targeting multi drug-resistant strains, there are numerous drugs in the (pre)clinical pipeline (website newtbdrugs.org), although monoclonal antibodies are still quite scarce. In 2012 there has been a clinical registry (NCT01638520) for pascolizumab, an anti-IL-4 antibody, a phase 2 study which was looking at the safety and efficacy in patients receiving standard therapy for pulmonary tuberculosis but the status is currently unknown and there has not been a subsequent publication in literature.

### Influenza and COVID-19

Both influenza and COVID-19 are respiratory viral diseases which can be contracted seasonally without a travel history. Although Influenza was found to be in the top-10 diseases seen in the returning traveler presetting with symptoms to the European travel clinic between 1998 and 2018, it is an endemic disease in almost all countries worldwide. There are yearly new vaccines available based on current strains for both diseases and administered to people with a higher risk of developing more severe disease such as the elderly or immunocompromised. For both influenza virus and SARS CoV-2 virus well written reviews on mAbs targeting these viruses have been recently published in literature and were therefore left out of this scoping review [86, 87].

### Other infections

The only licensed mAbs targeting bacteria causing tropical infections include raxibacumab and obiltoxaximab, used as post-exposure prophylaxis or treatment for inhalation anthrax (Table 1). (Pre)clinical studies on mAbs directed against (parts of) bacteria causing tropical infectious diseases are scarce, presumable due to effective antibiotic treatment with high cure rates against diseases such as leptospirosis, typhoid fever, and rickettsial disease (Table 2) although multi-drugs resistant bacteria causing these diseases are an increasing threat to global health [88]. Monoclonal antibodies targeting tropical bacterial infections with a high mortality rate despite antibiotic treatment such as melioidosis would be desirable and constitute a potential area of further research, but only preclinical studies have been reported (Supplementary file). Most parasitic infections caused by nematodes such as *Strongyloides stercoralis*, although having a high global burden, are not yet being targeted with mAbs in literature (Table 2). On the other hand, mAbs targeting both cutaneous as well as visceral leishmaniasis caused by the *Leishmania* parasites, have been studied preclinically over the last years (Supplementary file), and there is even a mAbs in de clinical stage targeting IL10 (anti IL-10, SCH708980, NCT01437020) which may help to prevent the immune system from becoming suppressed and worsening the disease in combination with standard therapy.

## Conclusion and future perspective

The use of immunoglobulins as (preventive) treatment strategy against infectious diseases have a long-standing history. Development of mAbs for (non-infectious and) infectious disease applications has evolved into one of the most dynamic fields in therapeutics development today. In the field of infectious diseases, in any case since the beginning of the COVID-19 pandemic, the pharmaceutical industry seems to put all its effort in the (pre) clinical development of these mAbs, with no expenses spared [18].

The increasing use of mAbs for preventive and curative purposes shall lead to more pressure on healthcare systems and especially higher costs. Ethical questions arise whether asymmetrical use as a luxury to be affordable only for travelers from non-endemic areas is desirable whereas patients in endemic areas will be deprived from potential benefits for mainly cost reasons; or should resources be devoted completely to fight infectious (tropical) diseases on a global scale. One could argue this is comparing apples with pears and both, developing treatment strategies for travelers and concurrently working on the eradication of diseases with a high burden in endemic countries, could go hand in hand. Using both preventive and therapeutic mAbs targeting infectious diseases in endemic areas would greatly reduce the burden (see examples of mAbs created against *P. falciparum* malaria). However, understanding the various barriers in healthcare systems that prevent patients from getting medicines they need is critical to establishing a global operations strategy for these mAbs [89]. Barriers such as product pricing, patient insurance, regulatory approval delays, prescribing practices, funding uncertainty and inefficient supply chain could prevent patients from receiving reliable access to monoclonal antibodies, especially in low- and middle-income countries (LMIC). Operation goals for essential medicines are informed by the WHO and should be affordable, available, and accessible. In the past, leveraging economies of scale has been key to greatly expanding the global affordability, accessibility and availability of life-saving vaccines and antiretroviral small molecule drugs. Successful introduction of mAbs will require a similar high-volume, low-cost operations strategy before implementation. For example, it was calculated that the seasonal administration of extended half-life mAbs immunoprophylaxis targeting RSV at birth in children from Mali would prevent 1300 hospitalizations, 31 deaths, and 878 disabilities-adjusted life-years (DALYs) for children through the first three years of life. Using these extended half-life mAbs as part of the preventive strategy was shown to be the optimal next-generation strategy for RSV lower respiratory tract infection (LRTI) prevention in Mali, if the product were to be priced similarly to routine pediatric vaccines, which depends on many factors [90]. Process and operations strategies to enable global access to antibody therapies have been reviewed in detail by Kelley et al. [89].

When mAbs are used as therapeutic option for travelers; then, the cost–benefit ratio could be more optimistic as these mAbs are mostly targeting life threatening or severely debilitating diseases such as rabies, yellow fever and EVD, and when administered timely, could lead to significant reduction in patient mortality and cost in terms of cutting down on duration of hospitalizations. For travelers, the use of a single dose of extended half-live mAbs against malaria preventing disease for three consecutive months would be preferable compared to a daily dose of malaria chemoprophylaxis if it would also outweigh the costs. The cost of mAbs in high income countries are often dependable on price agreements negotiated by the governmental bodies with pharmaceutical companies and are therefore difficult to determine up front. Although preventive treatment strategy vaccines are most likely less costly than mAbs for the immunocompetent traveler, this group of travelers have much to gain from mAbs similar to the infants receiving RSV mAb as immunoprophylaxis at birth when the immune system has not been fully developed [90]. Luckily, production efficiency of mAbs has increased dramatically over recent decades, and cell-culture expression levels around 4 g/l or even higher are common [91]. A recent estimate which—depending on process and volume—range from US$20/g to US$80/g and could render mAbs product pricing more affordable across settings and applications [92].

If affordable, a wide range of mAbs applications to fight ‘tropical’ infectious diseases, or better infectious diseases in low-and middle-, and high-income countries alike; applications in returning travelers should pave the way for ubiquitous access, where indicated, to roll out mAbs to fight infectious diseases globally.

### Supplementary Information

**Additional file 1:** **Supplementary file.** Contains the PMID/NCT identifiers of all the article which have been found during the search on PubMed and Clinicaltrial.gov respectively in an excelsheet.

## Data Availability

All data generated or analyzed during this study are included in this published article [and its supplementary information files].

## Abbreviations

mAbs: Monoclonal antibodies
COVID-19: Coronavirus disease 2019
EVD: Ebola virus disease
PEP: Post-exposure prophylaxis
HAV: Hepatitis A virus
HBV: Hepatitis B virus
HRIG: Human rabies immunoglobulin
CPT: Convalescent plasma therapy
WHO: World Health Organization
Ig: Immunoglobulins
Fabs: Fragment antigen-binding domains
Fc: Crystallizable fragment
FcyRs: Fcy receptors
RSV: Respiratory syncytial virus
ADE: Antibody-dependent enhancement
EMA: European Medicines Agency
FDA: U.S. Food and Drug Administration
PCR: Polymerase chain reaction
S.: *Salmonella* Spp
E.: *Escherichia* Spp
V.: *Vibrio*
C.: *Clostridioides*
PrEP: Pre-exposure prophylaxis
HIV-1: Human immunodeficiency virus type 1
PMID: Pubmed identifier
ZVD: Zika virus disease
IV: Intravenous
DMAbs: DNA-encoded monoclonal antibodies
CHIKV: Chikungunya virus
RNA: Ribonucleic acid
JEV: Japanese encephalitis virus
IVIG: Intravenous immunoglobulin
WNV: West Nile virus
EBOV: Ebola viruses
GP: Glycoprotein
SUDV: Sudan strain
HCC: Hepatocellular carcinoma
HCV: Hepatitis C virus
SVR: Sustained virological response
DAA: Direct-acting antiviral agent
CTLA: Cytotoxic T-lymphocyte-associated antigen
P.: *Plasmodium*
PfCSP: *P. falciparum* Circumsporozoite protein
TRA: Transmission-reducing activity
T: *Trypanosoma* Spp
S: *Schistosoma* Spp
IL: Interleukin
LMIC: Low- and middle-income countries
DALY’s: Disabilities-adjusted life-years
LRTI: Lower respiratory tract infection
ADA-SCID: Adenosine deaminase severe combined immunodeficiency
DAT: Diphtheria antitoxin
HBAT: Heptavalent botulism antitoxin
OS: Orphan drug status
Crimean Congo HF: Crimean Congo hemorrhagic fever
